# Hypothalamic orexinergic neurons modulate pain and itch in an opposite way: pain relief and itch exacerbation

**DOI:** 10.1186/s12576-022-00846-0

**Published:** 2022-08-22

**Authors:** Tatsuroh Kaneko, Tomoyuki Kuwaki, Hideki Kashiwadani

**Affiliations:** Department of Physiology, Graduate School of Medical and Dental Sciences, Kagoshima University, Kagoshima, 890-8544 Japan

**Keywords:** Orexin neuron, Lateral hypothalamus, Pain, Itch

## Abstract

Pain and itch are recognized as antagonistic sensations; pain suppresses itch and inhibition of pain generates itch. There is still a lack of evidence about the neural mechanism of the interaction between pain and itch in the central nervous system. In this study, we focused on the orexin (ORX) neurons in the lateral hypothalamus (LH), which mediate various “defense responses” when animals confront stressors. We found that the scratching behaviors induced by the pruritogen were significantly suppressed in ORX-neuron-ablated (ORX-abl) mice. The exaggerated pain behavior and attenuated itch behavior observed in ORX-abl mice indicated that ORX neurons modulate pain and itch in an opposite way, i.e., pain relief and itch exacerbation. In addition, most of the ORX neurons responded to both pain and itch input. Our results suggest that ORX neurons inversely regulate pain- and itch-related behaviors, which could be understood as a defense response to cope with stress environment.

**Supplementary Information:**

The online version contains supplementary material available at 10.1186/s12576-022-00846-0.

## Background

Both pain and itch are discomfort sensations, but trigger distinct defense responses to protect our body from external stressors. Pain evokes a withdrawal response to avoid tissue damage, whereas itch induces scratching behavior to remove irritants from the skin surface. It is well known that there are complicated interactions between pain and itch [1]. The noxious input generated by scratching relieves the itch sensation [2], thus pain can suppress itch. In line with the idea, the inhibition of pain processing by spinal opioids can generate itch [3].

The neural mechanisms underlying the interaction between pain and itch have been unveiled at the level of the spinal cord [4]. In the spinal cord, two models have been presented and discussed: labeled-line theory [5–9] and leaky gate model [10].

In contrast to the progress of research about the interaction between pain and itch in the spinal cord, there are few reports so far which show the possible contribution of supraspinal structures to the pain–itch interaction. Recent studies have shown the importance of periaqueductal gray (PAG), an evolutionarily conserved structure in the midbrain, in the regulation of pain and itch sensations [11, 12]. Activation of PAG glutamatergic neurons leads to enhanced itch and decreased pain behaviors, while inhibition of PAG glutamatergic neurons attenuates itch and potentiates pain [13, 14].

Meanwhile, there are no reports so far which examine the neural mechanisms of interaction between pain and itch in the upper central nervous system (CNS). To explore the neural circuit mechanism of the pain and itch interaction in the upper CNS, we focused on the orexin-producing neurons in the lateral hypothalamus.

The orexin neuropeptides A and B (also known as hypocretin-1 and -2) are derived from the precursor peptide prepro-orexin [15]. The ORX neural system plays an important role in various physiological processes, such as arousal, reward-seeking behavior, energy homeostasis, sensory modulation, stress processing, cognition, endocrine functions, and pain modulation [16]. Among these various roles of ORX neurons, we have specifically focused on the defense response against stressors [17, 18]. Defense response leads our body to prepare for the fight-or-flight behavior when facing stressors, e.g., autonomic changes such as increasing blood pressure, heart rate, respiration, and analgesia against pain stress. ORX neurons in LH act as a master switch to activate multiple efferent pathways of the defense response.

On a historical premise, LH has been suggested to be one of the pain modulation sites [19–21]. Recent evidence has also suggested that ORX neurons in LH play an important role to induce an analgesic effect as a defense response to pain by affecting their receptors within several specific brain areas including the locus coeruleus (LC) [22], ventral tegmental area (VTA) [23], PAG [24, 25], and spinal cord [26].

Itch is also a stressful sensation evoking a defense response, such as the scratching behavior, to remove irritants from the skin. In addition, previous research showed the possibility of involvement of LH in itch neural processing [27]. Therefore, it is speculated that ORX neurons in LH also have some functions in part of itch regulation, not only in pain regulation, to induce the defense response against external irritants.

From these backgrounds, we hypothesized that ORX neurons may be involved in the regulation of itch-related behavior. To test our hypothesis, we identified the functional role of hypothalamic ORX neurons in itch regulation using orexin mutant mice. Furthermore, we revealed the opposite roles of ORX neurons in pain and itch neural processing. Our results gave a new explanation of how pain and itch are regulated in the CNS.

## Methods

### Animals

WT mice (C57BL/6, male 22–30 g, *n* = 63), ORX-abl mice (male, 22–28 g, *n* = 57), ORX-KO mice (male 24–35 g, *n* = 12), and *cFos-tTA*; *TetO-GCaMP6* mice (male 29–35 g, *n* = 10) were used in this study. All animals were 8–16 weeks old at the start of each experiment. The generation of mutant mice has been described in detail elsewhere [28–32]. Regarding the method for selective ablation of orexin neurons (ORX-abl), orexin–tetracycline transactivator (tTA) mice, which express tTA exclusively in orexin neurons under the control of the human prepro-orexin promoter, were bred with tetO diphtheria toxin A fragment (DTA) mice (B6.Cg-Tg (tetO DTA) 1Gfi/J, The Jackson Laboratory) to generate orexin-tTA; tetO DTA mice. In these double-transgenic mice, doxycycline is removed from their chow starting from birth so by 4 weeks of age, almost all (> 97%) of orexin neurons are ablated. Mutant mice were maintained as heterozygotes and crossed to obtain null mutants. We backcrossed the mutant mice with C57BL/6 mice (Clea Japan Inc., Tokyo, Japan) for more than 10 generations. We have confirmed that the expression of orexin peptide disappeared in ORX-KO and ORX-abl mice [31, 33].

c*Fos-tTA*; *TetO-GCaMP6* mice were generated by crossing Tg(Fos-tTA, Fos-EGFP*)1Mmay mice (obtained from The Jackson Laboratory, Stock No: 018306) with *TetO-GCaMP6* knock-in mice (*B6;129-Actb*<*tm3.1(tetO-GCaMP6)Kftnk* >, obtained from the RIKEN Bio Resource Research Center, RBRC09552). The double-transgenic mice were confirmed via PCR.

Animals were housed with lights on at 7:00 am and off at 7:00 pm. All experiments were performed during the light cycle, i.e., between 10:00 am and 6:00 pm. All experiments were performed in accordance with the guidelines outlined by the Physiological Society of Japan and were approved by the Experimental Animal Research Committee of Kagoshima University (MD19102, MD19104).

### Drugs

Chloroquine diphosphate salt and capsaicin were purchased from Sigma-Aldrich. Chloroquine was dissolved in physiological saline and used freshly. Capsaicin was dissolved in physiological saline containing 7% Tween-80 (polyoxyethylene sorbitan monooleate, Wako) and used freshly.

### Behavioral assessment of itch (neck model)

Mice were handled at least 5 days before performing the behavioral experiments. All animals were acclimatized to the observation chamber for 3 days before the behavioral experiments. The pruritogen-induced neck-scratching test was performed as described previously [34]. The nape of the neck of mice was shaved the day before the experiment. The animals were placed individually in the observation chamber and allowed to habituate to it for 30 min. Chloroquine (200 μg/50 μL) was injected intradermally into the nape of the neck, and the mice were immediately placed into the observation chamber. Subsequently, the scratching behaviors were video recorded at 60 frames/s for 30 min in an unmanned environment, and the video was then played back to assess the scratching behavior. The scratching behavior was quantified by counting the number of scratching bouts, which consisted of one or more rapid back-and-forth hind-paw motions on the intradermal injection site. Counting of scratching behavior was performed in a blinded manner. The duration of scratch bouts and scratch frequency in each bout was not analyzed in this study.

### Behavioral assessment of pain and itch (cheek model)

The cheek model was performed as described elsewhere [35, 36]. Mice were handled at least 5 days before performing the behavioral experiments. All animals were acclimatized to the observation chamber for 3 days before the behavioral experiments. The right cheek of the mice was shaved the day before the experiment. Animals were moved to the recording cage for 30 min to acclimatize to the recording conditions. The mouse was then gently handheld, and the test substance (200 μg/30 μL of chloroquine or 40 μg/20 μL of capsaicin) was injected intradermally into the right cheek. Subsequently, the scratching and wiping behaviors were video recorded for 30 min in an unmanned environment, and the video was then played back to assess the wiping or scratching behavior.

To assess the itch-related behavior, we observed the scratching bouts over the injecting site. Typically, the test mouse showed a sequence of behaviors, i.e., raising a hind paw toward the cheek, scratching the cheek several times within a few seconds, and putting the paw down. Therefore, we defined this series of behaviors as a scratching bout, and the number of scratching bouts was counted. To assess the pain-related behavior, we counted the number of wiping of the injecting site. To distinguish the pain-related wiping (typically wiping of the injection site using the ipsilateral forepaw) from grooming (typically wiping using both forepaws), we counted the number of wiping events using a single ipsilateral forepaw. All analyses of behavioral assessment were performed in a blinded manner.

### Immunohistochemistry

Two hours after the chloroquine or saline injection, the mice were deeply anesthetized with urethane (1.3 g/kg, i.p.) and transcardially perfused with saline followed by 4% paraformaldehyde in 0.01 M PBS (pH 7.4). The brain was then excised, post-fixed at 4 °C overnight, and cryoprotected with 30% sucrose in 0.01 M PBS. Subsequently, 30-μm sections were prepared on a cryostat (Microtome Cryostar NX70, Thermo Fisher Scientific). Every alternate section was collected, and immunohistochemical staining of floating sections was performed. The sections were incubated with PBS containing 0.3% Triton-X and 1% normal horse serum for 30 min and then allowed to react with a rabbit anti-c-Fos monoclonal antibody (9F6, 1/1000, Cell Signaling Technology) overnight at 4 °C. After rinsing with PBS, the sections were incubated with secondary antibodies (CF488-conjugated anti-rabbit IgG, 1/500, Biotium) for 90 min in a dark box at room temperature (RT). The sections were then rinsed with PBS and reacted with a goat anti-orexin A polyclonal IgG (SC-8070, 1/200, Santa Cruz Biotechnology) for 1 h at RT. After rinsing with PBS, the sections were incubated with secondary antibodies (CF647-conjugated anti-goat IgG, 1/200, Biotium) for 1 h at RT. The sections were then mounted on a glass slide and examined under a fluorescence microscope (BZ-X700, KEYENCE, Osaka, Japan). Of note, we counted the number of c-Fos-positive cells within orexin-immunoreactive cells which were identified as ORX neurons.

Regarding the c-Fos immunohistochemistry in the PAG area, after the c-Fos staining, the sections were stained with NeuroTrace 640/660 deep red (1/100, Thermo Fisher Scientific, Waltham, MA, USA) for fluorescent Nissl staining to identify the PAG area. We counted the number of c-Fos-positive cells in the lateral part of PAG (lPAG) + ventrolateral part of PAG (vlPAG) and represented the density of c-Fos-positive cells (/μm^2^).

### Orexin neuron population experiment using c*Fos-tTA: TetO-GCaMP6* mice

c*Fos-tTA; TetO-GCaMP6* mice were fed doxycycline (DOX)-added chow (200 mg/kg) and water (700 mg/L) for 2 weeks, to reduce the baseline expression of GCaMP6. Subsequently, the animals were maintained under DOX(−) conditions for 2 days, to remove DOX from the body, and the algogen (40 μg/20 μL of capsaicin in physiological saline containing 7% Tween-80) was injected into the right cheek. Ten hours after the algogen injection, mice were returned to the DOX (+) condition for 2 days, to stop the de novo expression of GCaMP6. Thereafter, mice were injected with the pruritogen (200 μg/30 μL of chloroquine in physiological saline) into the right cheek. Two hours after the pruritogen injection, mice were deeply anesthetized with urethane (1.3 g/kg, i.p.) and transcardially perfused with Ringer’s solution (containing 3 mM CaCl_2_), followed by 4% paraformaldehyde in 0.1 M Tris (pH 7.4) + 3 mM CaCl_2_. We added calcium to the ordinal washing and fixative solutions because we found in our preliminary experiment that the fluorescence of GCaMP6 was better preserved in the presence of supplementation with calcium. The brain was excised and cryosectioned for immunohistochemistry. Sections were incubated with PBS containing 0.3% Triton-X and 1% normal horse serum for 30 min and then allowed to react with a rabbit anti-c-Fos monoclonal antibody (9F6, 1/1000, Cell Signaling Technology) overnight at 4 °C. After rinsing with PBS, the sections were incubated with secondary antibodies (CF750-conjugated anti-rabbit IgG, 1/500, Biotium) for 90 min in a dark box at RT. The sections were then rinsed with PBS and reacted with a goat anti-orexin A guinea pig anti-serum (389004, 1/500, Synaptic systems) for 1 h at RT. After rinsing with PBS, the sections were incubated with secondary antibodies (CF568-conjugated anti-guinea pig IgG, 1/500, Biotium) for 1 h at RT.

### Statistical analyses

Statistical analyses were performed via an unpaired *t*-test or two-way ANOVA with post hoc Tukey’s multiple comparisons test using the Prism9 software (GraphPad Software, San Diego, CA, USA). Statistical significance was set at *P* < 0.05 in all analyses.

## Results

### ORX neurons are activated by intradermal injection of a pruritogen

**Fig. 1:**
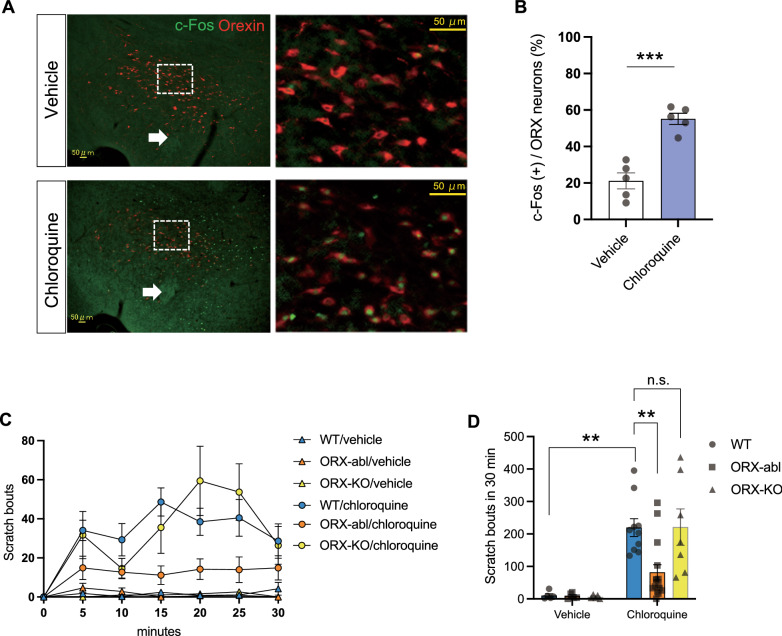
Orexin neuron ablation mice, but not orexin peptide-deficient mice, showed the decrease of pruritogen-induced scratching behavior. **A** c-Fos expression (green) in orexin neurons (red) in the lateral hypothalamus after intradermal injection of vehicle and chloroquine. **B** The quantification of c-Fos expression showed a significant increase in c-Fos-positive cells in orexin neurons (%) after the chloroquine treatment compared with the vehicle control. *n* = 5 for each group. **C** Time course of the number of scratching behaviors induced by intradermal chloroquine injection. The plots indicate the cumulative number of scratching bouts recorded every 5 min. **D** The scratching behavior within 30 min after chloroquine injection was significantly decreased in ORX-abl mice (*n* = 15), but not in ORX-KO mice (*n* = 7), compared with WT mice (*n* = 10). WT, wild-type mice; ORX-abl, orexin-neuron-ablated mice; ORX-KO, orexin-peptide-deficient mice. The arrows in **A** indicate fornix. The white boxed area in **A** left is magnified in **A** right. The data represent the mean ± SEM. ***P < 0.001 (unpaired *t*-test). **P < 0.01 (two-way ANOVA with post hoc Tukey’s multiple comparisons test)

As a first step to assess the involvement of the ORX system in itch sensation/regulation, we examined the response of ORX neurons to pruritic stimulation using c-Fos as a neuronal activity marker. Immunohistochemical analyses revealed that the ratio of c-Fos-positive ORX neurons to the total ORX neurons was significantly increased after intradermal injection of chloroquine into the nape of the neck (*R*_VEH_ = 21.2% ± 5.5%, *R*_CHL_ = 55.1% ± 10.4%, *P* < 0.001; Fig. 1A, B). This result indicates the contribution of the ORX system to the neural processing of itch.

### Orexin neuron ablation mice, but not orexin peptide-deficient mice, showed the decrease of pruritogen-induced scratching behavior

Next, to assess the contribution of ORX neurons to the neural processing of itch, we examined pruritogen (chloroquine)-induced scratching behaviors in two orexin mutant mice, ORX-abl mice, and ORX-KO mice. A time course analysis of the number of scratching bouts showed that the scratching behavior was attenuated in ORX-abl mice compared with their wild-type (WT) counterparts (Fig. 1C). In contrast to our prediction, the scratching behavior was not attenuated in ORX-KO mice. A two-way ANOVA with Tukey’s multiple comparisons test revealed that the number of scratching events recorded during the observation period of 30 min was significantly decreased in ORX-abl mice (Scratch_WT_ = 219.6 ± 27.2, Scratch_abl_ = 81.7 ± 23.4, Scratch_KO_ = 221.4 ± 56.0, *p*_WT/abl_ = 0.005, *p*_abl/KO_ = 0.014, *p*_KO/WT_ > 0.9999; Fig. 1D). These results indicate that ORX neurons contribute to the neural processing of the itch-induced scratching behavior, whereas the ORX peptide itself is unnecessary for this processing; other co-transmitter(s)/neuro-modulators expressed in ORX neurons may play a pivotal role in this processing.

### ORX neurons inversely modulate pain and itch sensations: pain relief and itch exacerbation

**Fig. 2:**
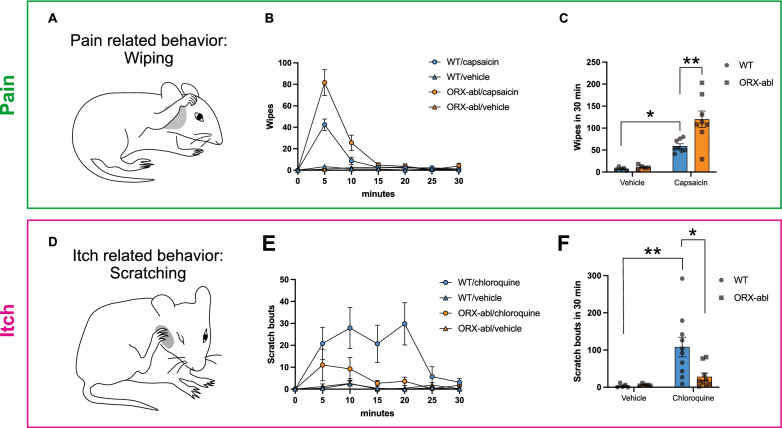
Orexin neurons modulate pain and itch sensations in an opposite way. **A** Illustration of the wiping behavior using a single forelimb (pain-related behavior). **B** Time course of the number of wiping behaviors induced by capsaicin injection into the cheek. The plots indicate the cumulative number of wiping events recorded every 5 min. **C** The number of wiping events within 30 min after capsaicin injection was significantly increased in ORX-abl mice (*n* = 8) compared with WT mice (*n* = 8). **D** Illustration of the scratching behavior using the hind leg (itch-related behavior). **E** Time course of the number of scratching bouts induced by chloroquine injection into the right cheek. The plots indicate the cumulative number of scratching bouts recorded every 5 min. **F** The number of scratching bouts within 30 min after chloroquine injection was significantly decreased in ORX-abl mice (*n* = 9) compared with WT mice (*n* = 10). The data represent the mean ± SEM. *P < 0.05, **P < 0.01 (two-way ANOVA with post hoc Tukey’s multiple comparisons test)

The behavioral itch test performed in ORX-abl mice indicated that ORX neurons contribute to the exacerbation of itch, which was in contrast to their reported role in pain relief [30, 37]. However, the marked functional difference in the response of the ORX neurons to the cutaneous stimulation could be caused by the differences in the stimulation site used, i.e., pain test performed on the foot or tail [30, 37] and itch test performed on the neck (current results). Therefore, we injected algogen (capsaicin) or a pruritogen (chloroquine) into the same site, i.e., the cheek [35, 36], and examined the pain-associated behaviors (wiping using the ipsilateral forelimb, Fig. 2A) and itch-associated behaviors (scratching using the hind limb, Fig. 2D).

In the cheek capsaicin test, the number of wiping events was significantly larger in ORX-abl vs. WT mice (Wipe_WT_ = 59.1 ± 5.1, Wipe_abl_ = 120.1 ± 18.8, *p*_WT/abl_ = 0.0038, Tukey’s multiple comparisons test; Fig. 2B, C). In contrast, the cheek chloroquine test revealed that the number of scratching bouts was significantly lower in ORX-abl vs. WT mice (Scratch_WT_ = 108.0 ± 26.5, Scratch_abl_ = 28.4 ± 10.0, *p*_WT/abl_ = 0.0157, Tukey’s multiple comparisons test; Fig. 2E, F). These results indicate that ORX neurons contribute to pain relief, but exacerbate the itch evoked at a given stimulation site.

### Most nociceptive ORX neurons are also activated by itch input

**Fig. 3:**
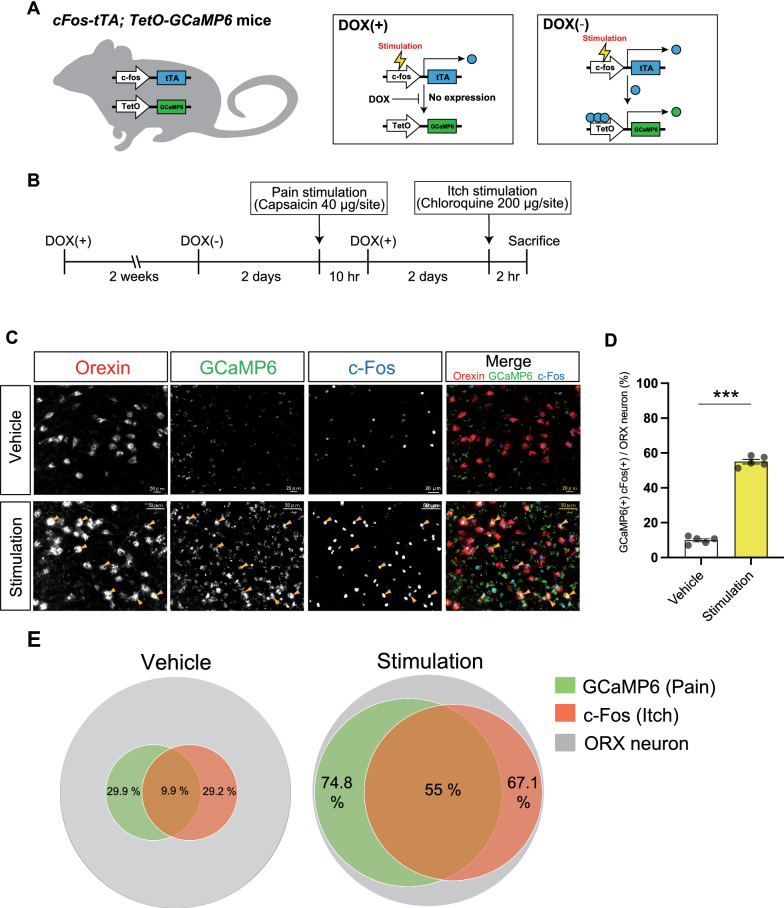
The same orexin neuron responded to both itch and pain stimulation. **A** Schematic diagram representing the temporally controlled expression of the GCaMP6 protein under *c-Fos* promoter using the Tet-off system in *cFos-tTA*; *TetO-GCaMP6* mice. **B** Diagram showing the experimental procedure, doxycycline timing, and schedule. **C** Expression of GCaMP6 (green) and c-Fos (blue) in orexin neurons (red) after the injection of vehicle or stimulants (capsaicin and chloroquine). Yellow arrowheads indicate the ORX cells overlapping with GCaMP6 and c-Fos expression. **D** The quantification of GCaMP6 and c-Fos expression in orexin neurons revealed a significant increase of GCaMP6 and c-Fos double-positive cells in orexin neurons (%) after pain and itch stimulation compared with the vehicle control. *n* = 5 for each group. The data represent the mean ± SEM. ***P < 0.001 (unpaired *t*-test). **E** Venn diagram showing the response ratio of orexin neurons to pain and itch stimulation. Left, the proportion of orexin neurons that responded to vehicle injection. Right, the proportion of orexin neurons that responded to pain (GCaMP6; green) and itch (c-Fos; red) stimulation. More than half of the orexin neurons responded to both pain and itch stimulation

We revealed that ORX neurons contribute to both pain relief and itch exacerbation, which raises the hypothesis that independent subpopulations of ORX neurons are responsible for these two functions. Alternatively, a subpopulation of ORX neurons may be responsible for both functions. To address these questions, we assessed the neuronal responses of ORX neurons based on the *c-fos* activity in *cFos-tTA*; *TetO-GCaMP6* mice (Fig. 3A). In the mutant mice, GCaMP6 expression is induced under the control of the *c-fos* promoter. Because the induction of GCaMP6 is restricted to the period during the doxycycline (DOX)-free condition, because of the use of the Tet-off system, we were able to select the time window of the induction. In our preliminary experiment, we confirmed that the fluorescence of GCaMP6 was well preserved after tissues were fixed with calcium-containing buffer and also confirmed that the GCaMP6 protein was retained within cells for more than 3 days after reversal to the DOX ( +) condition; thus, we were able to detect the activation of the *c-fos* promoter within a given time window via GCaMP6 expression. Concomitantly, the expression of the intrinsic c-Fos protein is induced by sensory stimulation and reaches a peak at 2–3 h after the stimulation [38], regardless of the presence of DOX.

Thus, we first injected the algogen (capsaicin) under DOX(−) conditions and induced the expression of GCaMP6. Two days later, we injected the pruritogen (chloroquine) under DOX(+) conditions and induced the expression of the intrinsic c-Fos protein (Fig. 3B). To minimize the effect of the difference in the receptive field of ORX neurons, we injected both drugs into the right cheek.

After the cheek test, both GCaMP6-positive (nociceptive) and c-Fos-immunopositive (pruriceptive) ORX neurons stimulated by capsaicin and chloroquine were increased compared with the vehicle control (from 29.9% to 74.8% of ORX neurons for pain stimulation (*p*_pain_ < 0.001, unpaired *t*-test, Additional file 1: Figure S1A), and from 29.2% to 67.1% of ORX neurons for itch stimulation [*p*_itch_ < 0.001, unpaired *t*-test, Additional file 1: Figure S1B)], indicating that the delivery of pain and itch stimulation to the cheek activated ORX neurons. The number of GCaMP6/c-Fos double-positive ORX neurons was also significantly increased, from 9.9% to 55% (*p*_pain/itch_ < 0.001, unpaired *t*-test, Fig. 3D, E). Note that the number of ORX neurons showing a specific response to pain or itch did not change (from 19.9% to 19.8% GCaMP6 ( +)/c-Fos( −) ORX neurons, *P* = 0.949, Additional file 1: Figure S1C; and from 19.3% to 12.1% GCaMP6( −)/c-Fos( +) ORX neurons, *P* = 0.126, Additional file 1: Figure S1D, unpaired *t*-test). The actual number of cell counting is presented in Additional file 2: Table S1. These results indicate that most ORX neurons respond to both noxious and pruritic stimulation within a given receptive field and may contribute to both pain and itch processing.

### Suppression of pruritogen-induced activation of PAG neurons in ORX-abl mice

**Fig. 4:**
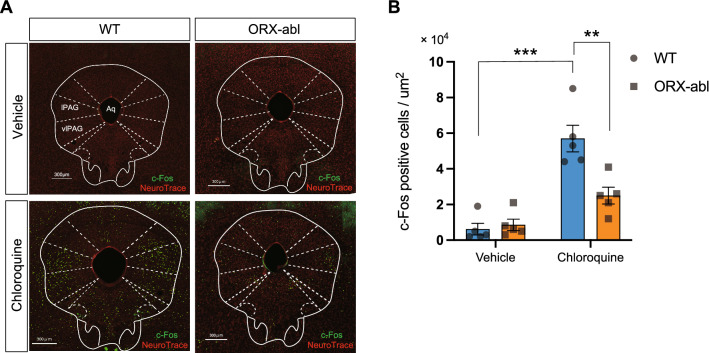
Attenuation of the pruritogen-induced activation of PAG neurons in orexin-neuron-ablated mice. **A** c-Fos expression (green) in lPAG/vlPAG neurons (red) after intradermal injection of vehicle or chloroquine into WT and ORX-abl mice. **B** The densities of c-Fos-positive neurons in the lPAG/vlPAG area after intradermal administration of vehicle or chloroquine were plotted. The c-Fos expression level after chloroquine injection was significantly decreased in ORX-abl mice compared with that in WT mice. The data represent the mean ± SEM. **P < 0.01, ***P < 0.001 (two-way ANOVA with post hoc Tukey’s multiple comparison test), *n* = 5 for all groups

The lateral and ventrolateral parts of the periaqueductal gray (lPAG and vlPAG, respectively) are key structures that modulate spinal itch processing via a descending pathway [14, 39]. Furthermore, the periaqueductal gray (PAG) is one of the major targets of ORX neurons [40, 41]. Therefore, we hypothesized that synaptic input from ORX neurons activates PAG neurons and facilitates the subsequent itch–scratching pathway. To address this hypothesis, we examined the neuronal activation of PAG neurons induced by intradermal injection of chloroquine into the nape using c-Fos immunohistochemistry. Figure 4A shows that the chloroquine injection-induced c-Fos expression in the lPAG in a WT mouse, whereas the expression level of c-Fos was not increased in an ORX-abl mouse. Tukey’s multiple comparison tests (Fig. 4B) revealed that the density of c-Fos-positive cells in lPAG + vlPAG was significantly increased in WT mice (*D*_WT-VEH_ = 0.63 × 10^4^/μm^2^, *D*_WT-CHL_ = 5.71 × 10^4^/μm^2^, *p*_WT(VEH vs. CHL)_ < 0.001), whereas that in ORX-abl mice was not significantly changed (*D*_abl–VEH_ = 0.87 × 10^4^/μm^2^, *D*_abl–CHL_ = 2.49 × 10^4^/μm^2^, *p*_abl (VEH vs. CHL)_ = 0.1312). Furthermore, the density detected after chloroquine injection was significantly smaller in ORX-abl mice compared with WT (*D*_WT–CHL_ = 5.71 × 10^4^/μm^2^, *D*_abl–CHL_ = 2.49 × 10^4^/μm^2^, *p*_CHL (WT vs. abl)_ = 0.0017). These results indicate that synaptic input from ORX neurons might be one of the major pathways underlying the activation of PAG neurons evoked by pruritic stimulation.

## Discussion

### ORX* neurons exacerbate pruritogen-induced scratching*

Here, we revealed for the first time that ORX neurons involve in the neural processing of itch. Based on the previous findings that ORX neurons are activated by noxious stimulations [42, 43], we revealed that ORX neurons were also activated by pruritic stimulation (Fig. 1A, B). In addition, we showed that the scratching behavior of ORX-abl mice was decreased compared with that of WT control mice after pruritogen stimulation (Fig. 1C, D). Therefore, our results suggest an involvement of ORX neurons in the neuronal processing of itch by exacerbating itch-related behavior, which could be understood as a defensive response aimed at removing irritants from the skin’s surface.

Regarding the physiological meaning of opposite regulation of pain and itch by ORX neurons, the concept of defense response could give an understandable explanation. Pain-stress induces analgesia to prepare for a fight-or-flight state, and Itch stress induces scratching behavior to remove irritants from the skin surface. Both of which could be understood as defense responses induced by ORX neurons to cope with stress conditions in our body.

### Parallel “pain relief” and “itch exacerbation” pathways are triggered by ORX neurons via orexin-dependent and orexin-independent transmission

Our cheek model experiment using ORX-abl mice suggests that ORX neurons modulate pain and itch sensations in an opposite way, i.e., pain relief and itch exacerbation (Fig. 2). In addition, based on the experiment performed using *cFos-tTA*; *TetO-GCaMP6* mice, we showed that more than half of the ORX neurons responded to both pain and itch stimulation (Fig. 3). These results support the hypothesis that most ORX neurons respond to both pain and itch stimulation, but modulate these two sensations in an opposite way. How could the same ORX neurons regulate the neural processing of pain and itch inversely?

The data included in Fig. 1 show that ORX-KO mice (orexin-peptide-deficient mice) did not exhibit an alteration of scratching behaviors compared with WT mice, in contrast to the significant inhibition of scratching behaviors observed in ORX-abl mice (ORX-neuron-ablated mice). These results indicate that the synaptic transmission by the orexin peptide is not important for the exacerbation of scratching behaviors; rather, co-transmitters/modulators expressed in ORX neurons, such as glutamate [44], dynorphin [45], or neurotensin [46], may play a dominant role in triggering the itch-exacerbate pathway. The ORX-dependent and ORX-independent function of ORX neurons has been reported in sleep regulation [47] and body temperature regulation [48]. Thus, in a similar mechanism, the ORX-independent function of ORX neurons might participate in the neural processing of itch. In contrast, several “pain-relief” pathways are triggered by orexin-dependent input from ORX neurons [25]. In addition to the direct suppression of the ascending pain pathway by ORX input [26, 49, 50], ORX neurons can drive the classical descending pain inhibitory pathway originated from the PAG via ORX-dependent synaptic transmission [37, 51]. Therefore, it is hypothesized that ORX neurons can drive both the “pain-relief” pathway via orexin-dependent transmission and the “itch-exacerbation” pathway via orexin-independent transmission. Further studies are needed to examine the hypothesis.

### Possible neural circuit mechanisms including hypothalamic ORX neurons for the regulation of pain and itch in an opposite way: projections to the PAG

Our results suggest that ORX neurons have inverse roles in pain and itch neural processing. ORX neurons in the LH project widely through various brain regions, such as the VTA, LC, dorsal raphe, and PAG [52]. Thus, we next addressed which neural circuit of ORX neurons is dominant in their inverse role in pain and itch regulation. The PAG was the candidate region because it plays inverse roles in the modulation of pain and itch neural processing [13, 14]. In pain regulation, a recent study demonstrated that the activation of glutamatergic neurons in the PAG suppresses nociception, whereas inhibition of glutamatergic neuronal activity potentiates nociception [13]. In itch regulation, the activation of glutamatergic neurons in the PAG leads to enhanced itch and decreased pain behaviors, whereas inhibition of PAG glutamatergic neurons attenuates itch but potentiates pain [14]. Our findings pertaining to the role of ORX neurons are in line with this observation. Anatomical studies have revealed that ORX neurons have a massive direct axonal projection to the PAG [40, 41]. Figure 4 shows that the itch-induced activation of PAG neurons was suppressed in ORX-abl mice, suggesting that the LH–PAG projection by ORX neurons may play a pivotal role in the exacerbation of the scratching behaviors induced by pruritogen injection. Gao and colleagues have demonstrated the existence of subpopulations of PAG glutamatergic neurons for dividing the function of modulating pain and itch, i.e., tachykinin 1 (Tac1)-expressing neurons and somatostatin (SST)-expressing neurons [39]. Tac1-positive glutamatergic neurons play an important role in facilitating the itch neural processing, because the ablation or pharmacogenetic inactivation of these neurons reduces itch-induced scratching behaviors. In contrast, SST-positive glutamatergic neurons do not affect itch neural processing. These findings together with our results led us to hypothesize that the orexin-independent synaptic input into ORX neurons activates Tac1-positive PAG glutamatergic neurons and triggers the itch-exacerbated pathway; in contrast, orexin-dependent synaptic transmission to other subpopulations of PAG glutamatergic neurons, e.g., SST-positive neurons, might play a dominant role in the pain-relief pathway. Further studies are needed to examine the neural circuit mechanism underlying the inverse control of pain and itch sensations by ORX neurons.

### Limitations of the experiments

Almost previous preclinical research on itch has used male animals [53], so our experiments only used male mice to reproduce the itch model as reported in previous studies. We consider conducting the experiments using female mice in a future study.

In the cheek model experiment (Figs. 2, 3), the vehicle which was used in pain- and itch-stimulation was different (pain: 7% Tween-80 in saline for capsaicin, itch: saline for chloroquine) because capsaicin is a water-insoluble agent. Therefore, the different vehicle injections might induce responses to a slightly different subpopulation of peripheral tissues and thus a slightly different subpopulation of orexin neurons in the LH.

In the ORX neuron population experiment using c*Fos-tTA; TetO-GCaMP6* mice (Fig. 3), the time resolution was of the order of hours. During the time window, the mice were presumably engaging in all sorts of movements/states such as eating, arousal, sleep, and so on. These movement/states are well-known to correlate with the activation of ORX neurons [54]. To minimize the effects of daily life on the c-Fos expression, we assessed the c-Fos expression induced by daily life under the vehicle injection and compared the expression between the vehicle group and the test group.

## Conclusion

We revealed for the first time that ORX neurons involve in the itch neural processing. Our data also suggest that ORX neurons modulate pain and itch sensations in an opposite way, i.e., pain relief and itch exacerbation. The present findings provide an explanation for how two related sensations, pain and itch, are controlled in the central nervous system.

### Supplementary Information

**Additional file 1: Figure S1**. Quantification of GCaMP6-positive, pain-responsive ORX neurons, and c-Fos-immunopositive, itch-responsive ORX neurons. **A** Quantification of GCaMP6 expression in orexin neurons after pain stimulation compared with the vehicle control. **B** Quantification of c-Fos expression in orexin neurons after itch stimulation compared with the vehicle control. **C** Quantification of GCaMP6-positive but c-Fos-negative cells in orexin neurons. **D** Quantification of GCaMP6-negative but c-Fos-positive cells in orexin neurons. *n* = 5 for each group. The data represent the mean ± SEM. ***P < 0.001 (unpaired *t*-test).

**Additional file 2: Table S1**. Cell counting number of GCaMP6-positive (pain-responsive) ORX neurons, c-Fos-immunopositive (itch-responsive) ORX neurons, and double-positive (pain and itch responsive) ORX neurons Cell counting number of ORX-positive, GCaMP6/ORX-positive, cFos/ORX-positive, and GCaMP6/cFos/ORX-positive cells. *n* = 5 for each group. The data represent the mean ± SEM.

## Data Availability

The datasets used and/or analyzed during the current study are available from the corresponding author upon reasonable request.
