# Supplementary figures and images for: Hypothalamic orexinergic neurons modulate pain and itch in an opposite way: pain relief and itch exacerbation
Source: J Physiol Sci. 2022 Aug 22;72:21. doi: 10.1186/s12576-022-00846-0 (PMC10717118; doi:10.1186/s12576-022-00846-0)

Figure S1.

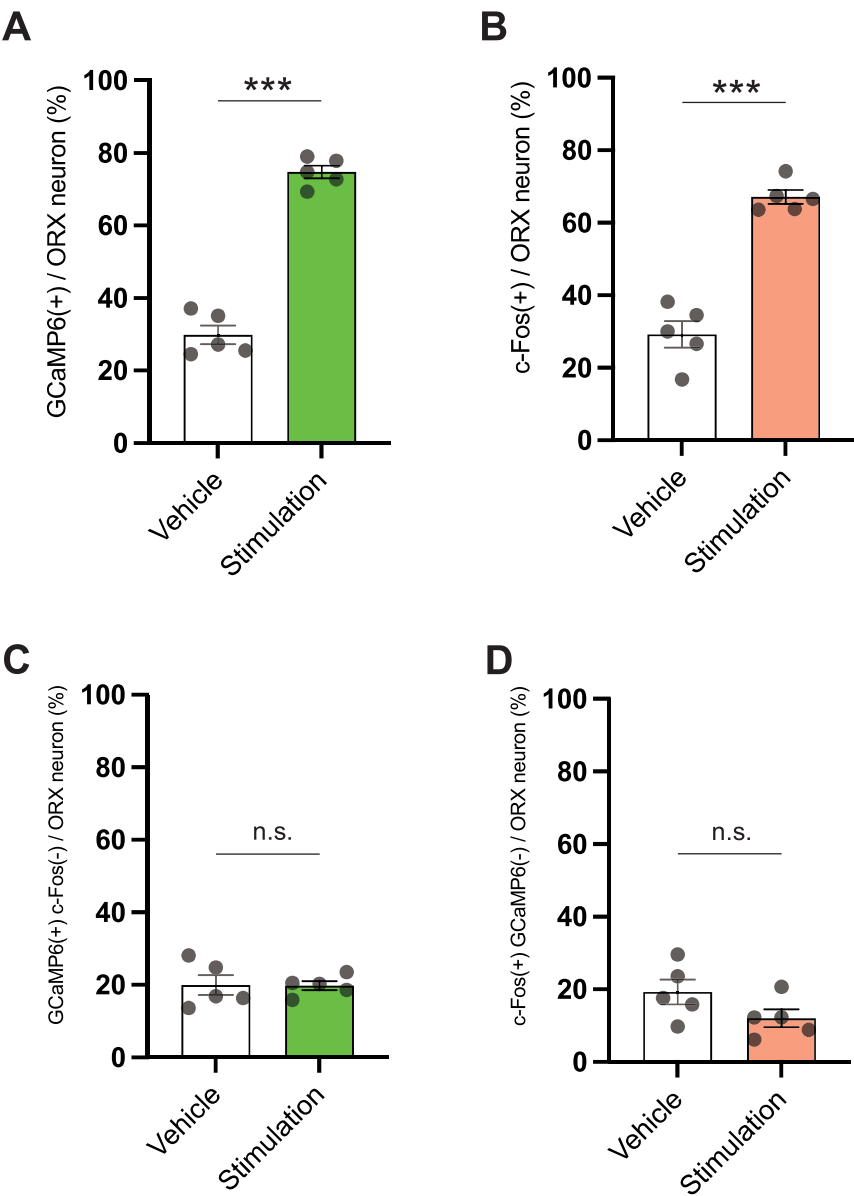

Supplement: Supplementary file 1 — Additional file 1: Figure S1. Quantification of GCaMP6-positive, pain-responsive ORX neurons, and c-Fos-immunopositive, itch-responsive ORX neurons. A Quantification of GCaMP6 expression in orexin neurons after pain stimulation compared with the vehicle control. B Quantification of c-Fos expression in orexin neurons after itch stimulation compared with the vehicle control. C Quantification of GCaMP6-positive but c-Fos-negative cells in orexin neurons. D Quantification of GCaMP6-negative but c-Fos-positive cells in orexin neurons. n = 5 for each group. The data represent the mean ± SEM. ***P < 0.001 (unpaired t-test). [file 12576_2022_846_MOESM1_ESM.pdf]
