# Supplementary material for: Hypothalamic orexinergic neurons modulate pain and itch in an opposite way: pain relief and itch exacerbation
Source: J Physiol Sci. 2022 Aug 22;72:21. doi: 10.1186/s12576-022-00846-0 (PMC10717118; doi:10.1186/s12576-022-00846-0)
Supplement: Supplementary file 2 — Additional file 2: Table S1. Cell counting number of GCaMP6-positive (pain-responsive) ORX neurons, c-Fos-immunopositive (itch-responsive) ORX neurons, and double-positive (pain and itch responsive) ORX neurons Cell counting number of ORX-positive, GCaMP6/ORX-positive, cFos/ORX-positive, and GCaMP6/cFos/ORX-positive cells. n = 5 for each group. The data represent the mean ± SEM. [file 12576_2022_846_MOESM2_ESM.pdf]

Table S1

Number of cells (in 3 slices / mouse)

	Vehicle (n=5)	Stimulation (n=5)
ORX(+)	100.4 ± 4.8	99.4 ± 4.9
ORX(+) GCaMP6(+)	26.4 ± 0.4	71.6 ± 1.8
ORX(+) c-Fos(+)	28.2 ± 5.6	63.9 ± 4.1
ORX(+) GCaMP6(+) c-Fos(+)	9.3 ± 1.4	51.8 ± 2.3
